# Seroprevalence of *Toxoplasma gondii* in Livestock and Poultry in Yunnan Province, China: A Cross-Sectional Study

**DOI:** 10.3390/vetsci13060517

**Published:** 2026-05-27

**Authors:** Meng-Ling Deng, Zhi-Yan Sun, Yu-Cong Zhang, Man-Li Jiang, Lu-Yang Wang, Jian-Fa Yang, Xing-Quan Zhu, Jun-Jun He, Feng-Cai Zou

**Affiliations:** 1 The Yunnan Key Laboratory of Veterinary Etiological Biology, College of Veterinary Medicine, Yunnan Agricultural University, Kunming 650201, China15681970721@163.com (M.-L.J.); wly952659965@foxmail.com (L.-Y.W.); jsc315@163.com (J.-F.Y.); 2 College of Bioengineering, Sichuan Water Conservancy Vocational College, Chengdu 611130, China; 3 Shanxi Key Laboratory of Animal Disease Research, Prevention and Control, College of Veterinary Medicine, Shanxi Agricultural University, Taigu 030801, China; xingquanzhu1@hotmail.com

**Keywords:** *Toxoplasma gondii*, livestock and poultry, modified agglutination test, seroprevalence, Yunnan Province

## Abstract

*Toxoplasma gondii* is a food- and environment-borne protozoan that is associated with human infection, food safety and animal health. Despite Yunnan Province being a major food-producing animal region in China, comprehensive data on the seroprevalence and risk factors of *T. gondii* infection in its food animals remain limited. This study investigated the seroprevalence of *T. gondii* infection among pigs, sheep and goats, cattle and poultry across all 16 prefectures/cities in Yunnan Province. From April 2023 to December 2024, a total of 10,766 blood samples were collected from clinically healthy livestock and poultry, including 2954 pigs, 1950 cattle, 1961 sheep and goats, and 3901 poultry. Sera were examined for the presence of specific antibodies against *T. gondii* by the Modified Agglutination Test (MAT). Seroprevalence was calculated, and statistical analyses were conducted to assess risk factors (geographic location, season, and animal species). The overall seroprevalence was 13.7% (1474/10,766), with species-specific rates of 26.3% (516/1961) in sheep and goats, 15.1% (446/2954) in pigs, 12.9% (252/1950) in cattle, and the lowest (6.7%, 260/3901) in poultry. Seroprevalence varied considerably across regions, ranging from 9.4% to 27.5%, with the highest prevalence rate being observed in Diqing (27.5%, 141/515). Based on statistical analysis, season, region and animal species were identified as significant risk factors associated with *T. gondii* infection in Yunnan Province. Overall, this first province-wide investigation revealed widespread exposure of *T. gondii* across both regions and species. Stringent and sustained control measures against toxoplasmosis of livestock, poultry, and humans in Yunnan Province are recommended.

## Simple Summary

*Toxoplasma gondii* is a zoonotic protozoan parasite that infects humans as well as a wide range of livestock and poultry, resulting in substantial economic losses and public-health risks. In this study, we investigated *T. gondii* seroprevalence and associated risk factors in livestock and poultry across 16 prefectures/cities in Yunnan Province, southwestern China. Seropositivity was detected in livestock and poultry from all 16 surveyed prefectures of Yunnan Province, indicating widespread exposure to *T. gondii*. *T. gondii* seroprevalence was highest in small ruminants (sheep and goats) and lowest in poultry. At the regional level, Diqing Prefecture had the highest seroprevalence among all prefectures surveyed. Season, region, and host species were identified as significant risk factors for *T. gondii* infection. Although the *T. gondii* prevalence observed in this study was much lower than those reported in previous studies, suggesting a downward trend over time, marked regional variation persists. Therefore, stringent and sustained control measures against *T. gondii* infection in livestock, poultry, and humans in Yunnan Province are recommended.

## 1. Introduction

*Toxoplasma gondii*, a food-borne, zoonotic protozoan parasite with worldwide distribution, is capable of infecting humans as well as a broad range of warm-blooded animals, particularly pigs, cattle, sheep, and goats [1]. Livestock and avian diseases constitute a public-health concern [2], as handling or eating contaminated raw or undercooked meat from infected animals is a principal route of pathogen transmission to humans [3,4,5]. Additionally, the consumption of infected raw milk may serve as a potential vehicle for *T. gondii* transmission [1]. It is generally assumed that approximately 30–50% of the human population is infected by this parasite [6]. Although toxoplasmosis is often subclinical in both humans and animals, vertical transmission or immunocompromised hosts can be fatal, causing abortion, stillbirth, congenital malformations and subsequent complications [7,8]. These adverse effects are common not only in humans but also in economically important livestock such as pigs and small ruminants. As farm animals represent a direct source of human infection [4,5], controlling *T. gondii* infections in livestock and poultry is of considerable importance.

Yunnan Province is located in Southwestern China, where its monsoon climate has endowed its high biodiversity. Additionally, Yunnan Province harbours numerous indigenous livestock breeds, including Diannan small-ear pigs, Yunling cattle, Ninglang black sheep, Yunnan semi-fine-wool sheep, Chahua chickens and Wuding chickens, that are crucial for local animal-production systems. Meanwhile, as a major livestock-producing region in China, Yunnan yields 5.058 million metric tons of meat from livestock and poultry in 2024 (https://stats.yn.gov.cn/Pages_23_6415.aspx, accessed on 1 May 2026). Hotpot, BBQ, and raw milk are favorite foods in Yunnan Province. In addition, some areas still retain the custom of eating raw meat. Unfortunately, *T. gondii* is not included in routine meat inspection protocols in China, leaving the food supply chain devoid of targeted preventive measures, an omission that may pose a public-health risk.

Serological survey is expected to provide information for assessing the possibility and potential frequency of the contact between the hosts and *T. gondii*, assisting the evaluation of the associated public-health risk posed by this zoonotic parasite. Preliminary seroprevalence surveys of *T. gondii* in humans and animals have been conducted in Yunnan Province by different methods and diagnostic kits. However, systematic, large-scale surveillance data delineating the prevalence landscape of *T. gondii* in livestock are still conspicuously absent, and the results can vary due to the applied methods and the scale of samples. Consequently, the present study is designed to determine the seroprevalence and risk factors of *T. gondii* infection in livestock and poultry across all 16 prefectures/cities of Yunnan with a single sensitive technique (Modified Agglutination Test, MAT).

## 2. Materials and Methods

### 2.1. Study Design and Samples Collection

**Figure 1:**
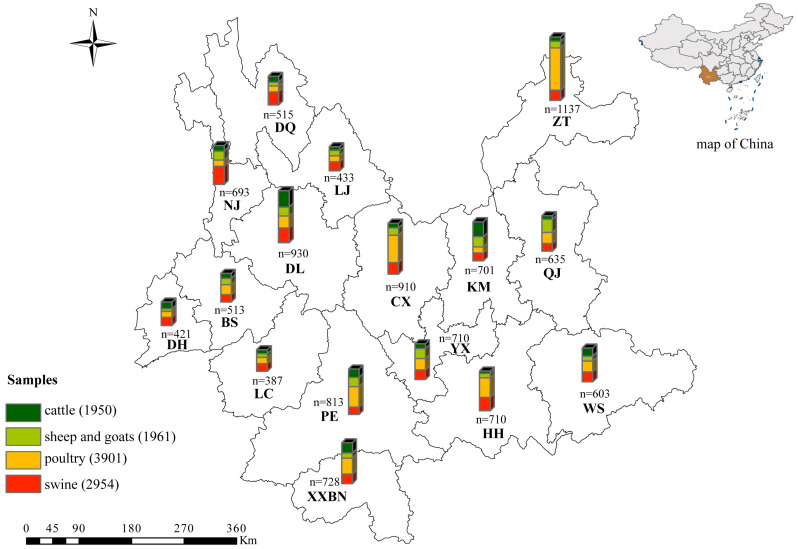
Sample collection sites for livestock and poultry serum samples across all 16 prefectures/cities in Yunnan Province. DQ, Diqing; NJ, Nujiang; LJ, Lijiang; DL, Dali; BS, Baoshan; DH, Dehong; LC, Lincang; PE, Pu’er; XXBN, Xishuangbanna; ZT, Zhaotong; CX, Chuxiong; KM, Kunming; QJ, Qujing; YX, Yuxi; HH, Honghe; WS, Wenshan. The n represents the number of samples of all species in one site.

From April 2023 to December 2024, a total of 10,766 serum samples were collected from pigs (n = 2954), cattle (n = 1950), sheep and goats (n = 1961), and poultry (mixed chickens and ducks, n = 3901) across 16 prefectures/cities of Yunnan Province, including Kunming, Qujing, Yuxi, Baoshan, Zhaotong, Lijiang, Pu’er, Lincang, Chuxiong Yi Autonomous Prefecture, Honghe Hani and Yi Autonomous Prefecture, Wenshan Zhuang and Miao Autonomous Prefecture, Xishuangbanna Dai Autonomous Prefecture, Dali Bai Autonomous Prefecture, Dehong Dai and Jing-po Autonomous Prefecture, Nujiang Lisu Autonomous Prefecture, and Diqing Tibetan Autonomous Prefecture (Figure 1). Detailed sampling information for each site, including sampling location and the number of samples collected from each livestock and poultry species, is provided in Supplementary Table S1. Samples were immediately placed in portable ice boxes and transported to the laboratory for detection of *T. gondii* antibodies.

### 2.2. Detection of T. gondii Antibodies

All serum samples were tested for *T. gondii* IgG antibodies by MAT, as previously described [9]. Sera were screened at dilutions 1:25, 1:50 and 1:100, and titers ≥ 1:25 indicate *T. gondii* infection. Formalin-fixed *T. gondii* RH tachyzoites were provided by the University of Tennessee (Knoxville, TN, USA). Positive and negative control sera were used.

### 2.3. Statistical Analysis

We categorized the samples by region, season, species, gender and age (age < 12 months and ≥12 months) for analysis of potential infection risk factors. IBM SPSS 27.0 software was used for statistical analyses. *T. gondii* seroprevalence was estimated from the ratio of positive samples to the total number of samples tested, and 95% confidence intervals (CI) were also given. Associations between *T. gondii* seroprevalence and the explanatory variables were screened using the Chi-square test. All statistical variables with a *p*-value < 0.05 were considered statistically significant.

## 3. Results

### 3.1. Comprehensive Seroprevalence and Risk Factors of T. gondii Infection in Livestock and Poultry in Yunnan Province

*T. gondii* antibodies were detected in 13.7% (1474) of the 10,766 serum samples collected from all four livestock groups across Yunnan’s 16 regions. Among the four food animals, small ruminants exhibited the highest exposure, with 26.3% (516/1961) of sheep and goats testing positive, followed by pigs (15.1%, 446/2954) and cattle (12.9%, 252/1950), whereas poultry had the lowest prevalence at 6.7% (260/3901). Species was a significant predictor of infection (*p* < 0.001); compared with poultry, the seroprevalence of *T. gondii* was higher in sheep and goats, pigs, and cattle (*p* < 0.001), with an odds ratio of 5.00, 2.49 and 2.08, respectively (Table 1).

Seasonal subgroup analysis revealed a bimodal pattern; the seroprevalence peaked in summer (16.7%, 155/927) and winter (19.3%, 561/2903), declined in autumn (11.4%, 431/3795) and was lowest in spring (10.4%, 327/3141). Compared with spring, the odds of seropositivity were 1.73 and 2.06 times higher in summer and winter, respectively (*p* < 0.001).

**Figure 2:**
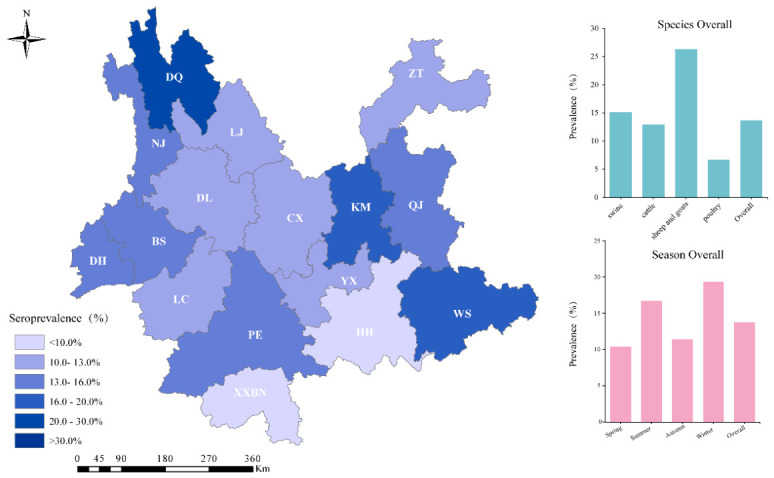
Overall prevalence of *T. gondii* in livestock and poultry by region, host species, and season. Abbreviations are identical to those defined in Figure 1.

Geographically, infection rates differed across different prefectures/cities (Table 1), and the geographical distribution map is shown in Figure 2. The highest seroprevalence was recorded in Diqing (27.4%, 141/515), followed by Wenshan (17.3%, 104/603), Kunming (16.5%, 123/744), Baoshan (15.8%, 81/513) and Pu’er (15.7%, 128/813), while the lowest infection rate was observed in Honghe (9.4%, 67/710). Xishuangbanna, Chuxiong, Dali, Lijiang, Lincang, Yuxi, and Zhaotong showed a similar prevalence within 10% to 12.3%. Notably, Diqing, an alpine pastoral region, displayed twice the provincial average; the risk of animals in Diqing being infected was 3.6-fold higher than Honghe (OR = 3.6, *p* < 0.001), implying altitude-related ecological drivers. Overall, the association between the seroprevalence and the geographic location was statistically significant (*p* < 0.01).

### 3.2. Seroprevalence and Risk Factors of T. gondii Infection in Pigs

The results showed that 446 out of 2954 (15.1%) were *T. gondii* IgG seropositive (Table 2). Anti-*T. gondii* antibodies were detected in all surveyed regions (Supplementary Table S2); the highest rates occurred in Diqing (33.1%, 77/233), followed by Dehong (18.2%), Zhaotong (18.1%), Kunming (17.6%) and Wenshan (16.4%). Diqing’s prevalence was 2.19-fold the provincial mean, whereas the lowest rate was recorded in Yuxi (7.6%, 13/171). Inter-prefecture differences were highly significant (*p* < 0.001), identifying geography as a major risk factor.

Seasonal distribution showed a pronounced bimodal pattern (Table 2). Prevalence peaked in summer (28.7%, 55/192) and winter (24.1%, 134/556), declined in spring (13.0%, 110/845), and reached its nadir in autumn (10.8%, 147/1361). The season was an independent risk factor for porcine toxoplasmosis in Yunnan Province.

There was no statistical difference in *T. gondii* seroprevalence by sex or age category of the tested animals.

### 3.3. Seroprevalence and Risk Factors of T. gondii Infection in Cattle

Among the 1950 bovine sera, 252 tested positive (12.9%, Table 2). Animals that tested positive were detected in every collection site, but exposure clustered geographically (Supplementary Table S2). Similar to pigs, the highest prevalence in cattle was also recorded in Diqing (20.0%, 22/110). Inter-prefecture differences were significant (*p* < 0.01), indicating that location is an important risk factor for bovine toxoplasmosis.

Neither sex nor age influenced infection risk. Seroprevalence was 14.0% in cows, 10.3% in bulls and 13.4% in animals of unrecorded sex (*p* = 0.294, Table 2). Similarly, no significant effect from age difference was observed: 10.5% in adults, 16.0% in juveniles, and 13.4% in animals of unknown age (*p* = 0.252).

Seasonal analysis revealed winter as the low-risk period (9.4%; 65/693; 95% CI: 7.43–11.78%), with a sharp rise in autumn (16.3%; 90/551; 95% CI: 13.48–19.65%); spring and summer showed intermediate values of 14.5% and 12.0% (Table 2), respectively. Using winter as a reference, season was an independent risk variable (χ^2^ = 31.62, df = 3, *p* < 0.001), with autumn results showing a strong association (OR > 3, 95% CI: 2.40–4.88).

### 3.4. Seroprevalence and Risk Factors of T. gondii Infection in Sheep and Goats

A provincial serosurvey of 1961 sheep and goat sera revealed a global *T. gondii* prevalence of 26.3% (516/1961, Table 2). Infection exhibited a clear altitudinal–climatic gradient (Supplementary Table S2): the north-eastern highlands (Zhaotong 41%, Wenshan 40%, Diqing 39%) registered the highest seroprevalence (OR 6.99–10.24 vs. reference), the southern hot-humid belt (Baoshan, Dehong, Xishuangbanna and Pu’er) formed an intermediate 30–40% zone (OR 3–5), while the central temperate plateau (Dali, Chuxiong, Kunming, Lijiang and Lincang) remained at 15–20%, with Dali showing the lowest seroprevalence (9.9%). Prefecture-level risk paralleled elevation, mean annual temperature and grazing intensity, indicating strong spatial aggregation.

The analysis of possible risk factors of *T. gondii* infection in sheep and goats showed that the regions and season were considered as important risk factors, but not sex or age. Ewes, rams and animals of unrecorded sex showed seroprevalences of 20.2% (45/223), 23.9% (81/339) and 27.9% (390/1399) (Table 2), respectively. Rams carried a 1.24-fold higher odds of infection than ewes (OR = 1.24; 95% CI: 0.82–1.87), whereas the unknown-sex group had a 1.53-fold excess risk (OR = 1.53; 95% CI: 1.08–2.16); the overall effect of sex was significant (*p* = 0.029).

Age stratification revealed a similar pattern: 20.2% (24/119) in lambs (<12 months), 23.0% (102/443) in adults (≥12 months) and 27.9% (390/1399) in sheep of unrecorded age. Compared with lambs, adults showed an OR of 1.18 (95% CI: 0.72–1.95) and the unknown-age group an OR of 1.53 (95% CI: 0.96–2.43); the age-associated difference was also statistically significant (*p* = 0.038).

Seasonal analysis disclosed a pronounced bimodal profile. Spring baseline prevalence was 18.7% (59/315; 95% CI: 14.81–23.41%), rose sharply to 36.7% (54/147; 95% CI: 29.37–44.77%) in summer, declined to 21.3% (146/685; 95% CI: 18.41–24.54%) in autumn and rebounded to 31.6% (257/814; 95% CI: 28.47–34.85%) in winter. Relative to spring, summer and winter carried 2.52-fold and 2.00-fold higher odds of infection, respectively, whereas autumn did not differ significantly (OR = 1.18; 95% CI: 0.84–1.65; *p* > 0.05).

### 3.5. Seroprevalence and Risk Factors of T. gondii Infection in Poultry

A total of 3901 poultry sera (mixed chickens and ducks) were collected, corresponding to an overall seroprevalence of 6.7% (95% CI: 5.92–7.49%) (Table 1). Using Xishuangbanna (3.5%; 10/288) as reference (Supplementary Table S2), the poultry in high-altitude prefectures displayed significantly higher *T. gondii* positive rates: 13.6% in Diqing (15/110; OR = 4.39; 95% CI: 1.91–10.10), 10.5% in Chuxiong (51/488; OR = 3.24; 95% CI: 1.62–6.50) and 11.1% in Kunming (12/108; OR = 3.48; 95% CI: 1.46–8.30). Inter-prefecture heterogeneity was highly significant, indicating that altitude-linked temperature gradients and management practices modulate oocyst survival and poultry exposure risk across Yunnan.

Males and females showed similar seroprevalences (5.0% vs 5.5%) (Table 2), whereas results for the unknown-sex group rose to 7.6%. Age exerted a significant effect (*p* = 0.014): birds < 90 d carried 10.4% positivity, while both 90–180 d and > 180 d groups stabilized at 5.2%. The effect of their production system was predictable: free-range flocks had 4.4% seroprevalence compared with 8.6% in large-scale confined farms (OR = 2.08; 95% CI: 1.53–2.83; *p* < 0.001), indicating that intensive husbandry amplifies exposure.

Seasonally, autumn recorded the lowest rate (4.01%; 48/1198; 95% CI: 3.04–5.27%) (Table 2). Winter showed a sharp increase to 12.50% (105/840; 95% CI: 10.43–14.91%), yielding an OR of 3.42 (95% CI: 2.40–4.88) relative to autumn. Spring and summer displayed intermediate risks (OR = 1.49 and 1.36, respectively). A χ^2^ trend test confirmed winter as a distinct high-risk period (*p* < 0.001).

## 4. Discussion

*T. gondii*, one of the most widespread zoonoses, infects humans via oocysts- or tissue cysts-contaminated foods [10], making livestock and poultry central to its food-borne transmission. Consequently, accurate epidemiological monitoring of *T. gondii* infection in domestic animals is imperative to inform evidence-based policies and to quantify both environmental oocyst contamination and the risk to human health. Hence, a systematic elucidation of *T. gondii* epidemiology is urgently needed in regions such as Yunnan Province, where livestock numbers are large and host assemblages are complex.

Among the available diagnostic approaches, molecular methods are prone to false negatives because tissue cysts exhibit focal distribution within hosts. Serological assays are therefore preferred for large-scale surveillance and monitoring in meat safety owing to their simplicity, low cost and broad species applicability. The MAT has been extensively employed to detect IgG antibodies to *T. gondii* in sera, and is the most widely validated serological tool for animals, wildlife and humans [11,12,13,14,15]. In the present study, we employed MAT to detect *T. gondii*—specific IgG antibodies in sera from livestock and poultry sampled across all 16 prefectures/cities of Yunnan Province, aiming to generate a robust estimate of parasite seroprevalence in these populations. Previous research have reported that the diagnostic method may be a source of heterogeneity [16,17,18]; we used MAT exclusively to estimate the exposure rate, thereby eliminating method-based variation.

This study provides the first provincial serological insights into toxoplasmosis in livestock and poultry in Yunnan Province. An overall seroprevalence of 13.7% was found, which was markedly lower than the 28.3% in food-producing animals during 2000–2019 [16]. Our results suggested that the exposure rate was much less common in Yunnan Province than previously reported. Here, the global *T. gondii* seroprevalence was 13.7%, compared with 15.3% (China, 2010–2023) [17] and 23.7% (China, 2000–2017) [19], respectively. It is also below the 21.8% recently described for southwestern China and the 24.7% previously estimated for Yunnan itself [17], indicating a declining provincial trend. Compared with other Chinese regions, the Yunnan estimate was lower than those of eastern (20.5%), northern (17.7%), and central China (15.9%), but similar to northeastern (10.5%), northwestern (12.4%), and southern China (13.7%). This reduction is probably attributable to the development of the intensive management and health management of the animal husbandry in China, which minimize oocyst contact [20]. In addition, contingencies related to COVID-19, African swine fever, and avian influenza (e.g., movement restrictions, heightened biosecurity, wildlife-protection enforcement) may have further interrupted the eco-epidemiological circulation of *T. gondii* between wildlife, cats, and food animals.

Host species emerged as the principal determinant of *T. gondii* seroprevalence in Yunnan. Yunnan is famous for its diversity in species, and is a hotbed for zoonotic diseases. The difference in *T. gondii* prevalence among different hosts could be attributed to variability in susceptibility [21], and the observed hierarchy: sheep and goats 26.3% > pigs 15.1% > cattle 12.9% > poultry 6.7%, mirrors species-specific susceptibility shaped by host immunity and parasite evasion strategies. Here, sheep and goats displayed the highest seroprevalence (26.3%), while poultry displayed the lowest prevalence (6.7%), and pigs and cattle displayed intermediate levels. This order was inverse of the nationwide hierarchy [17,22,23], in which pigs were the highest and cattle were the lowest, with sheep and goats in the mid-level. This pattern likely reflects the combination of Yunnan’s vertical climate, which fostered succulent shrubs that sustain extended outdoor grazing, and the prevailing practice of year-round tether-free herding, thereby maximizing the contact possibility of definitive (felids) and intermediate (rodents, wild birds) hosts with sheep and goats.

Small ruminants act as sentinels of environmental oocyst contamination because of their ground-level grazing. The 26.3% prevalence in sheep and goats was double that of the 2010–2023 national average (11.2%), and aligned with the 27.0% reported earlier for Yunnan [17]. In contrast, this prevalence was lower than that in Zhejiang (37.8%), Beijing (35.2%) and Hubei (31.7%) [24], where lower altitude, eastward river flow and higher pet cat density favoured oocyst accumulation and sporulation [19]. Another possible explanation was that higher disposable incomes in these affluent regions promoted cat ownership, while hot and humid climates accelerated oocyst sporulation; contamination of drinking water and pastures with feline faeces consequently drove the elevated *T. gondii* prevalence recorded in sheep and goats [25]. BBQ is a common way to eat lamb meat, which often fails to kill all pathogens in the meat. Hence, high infection rate in sheep and goats made them a source for human and animal infections [26,27].

Pigs, implicated in 30–60% of human acute toxoplasmosis through pork consumption [25], showed 15.1% seroprevalence, below both of China’s average infection in 2010–2023 (23.2%) [17] and the previous data of Yunnan (26.1%) [24], but higher than those reported in Qinhai (11.8%), Guangdong (12.0%), Ningxia (13.0%) and Helongjiang (10.5%) [24]. Epidemiological studies have linked porcine *T. gondii* infection to environmental oocyst shedding by felids, exposure to infected rodents, on-farm management practices, and long-distance pig transport [19,28]. Intensification of the provincial pig industry has evidently reduced exposure.

Although cattle exhibit greater resistance to *T. gondii*, the prevalence was 12.9%, which corresponds with Tonouhewa et al. [29] and Pereira et al. [30] who reported the lowest prevalence rate of *T. gondii* infections in cattle than in pigs and sheep. In China, the seroprevalence of *T. gondii* infection ranged from 0.75 to 30.34%; our result was lower than in Guizhou (30.3%), Zhejiang (27.6%) and Chongqing (27.3%) [24], and higher than in Hunan (8.3%) [29], Liaoning (6.0%) and Guangdong (5.7%) regions in China previously [31]. Moreover, higher prevalence had been reported in Israel (29.4%) [32] and South Africa (32.6%) [33], and lower seroprevalence rates had been reported in Brazil (2%) and Switzerland (3.8%) [34]. Similar to small ruminants, the predominant route of *T. gondii* transmission in cattle was the ingestion of sporulated oocysts disseminated on forage, feed, and in water sources [35]. Beef is another major meat consumed in Yunnan, and a substantial proportion of consumers eat it raw with the belief that this practice preserves nutrients and improves palatability. Therefore, the risk of *T. gondii* infection in humans may be increased by raw contaminated beef.

Notably, the 6.7% *T. gondii* prevalence detected in poultry here was markedly lower than the national average of 19.9% reported for chickens and the aggregated 30.5% [17] recorded across southwestern China, but consistent with a recent local survey [36]. A meta-analysis reported 21.1% prevalence in Yunnan chickens [37], underscoring a genuine downward trend, possibly linked to expanded commercial cage systems that limited contact with oocyst-littered soil. In Guangxi and Jiangxi, where the climate was warm and humid, conditions that favoured oocyst survival and sporulation, prevalence in chicken exceeded 35% [24]. Conversely, the low rates in Xinjiang and Gansu coincide with prolonged drought that limited oocyst viability and sporulation. Yet, contemporaneous surveys of chicken *T. gondii* prevalence in southwestern provinces, including Sichuan and Chongqing, reported higher [37] results than that observed in this study, suggesting that factors other than climate were responsible for the low *T. gondii* positivity recorded in the present study.

The seroprevalence for livestock and poultry showed that *T. gondii* infection was widespread in meat-producing animals in different regions of Yunnan Province, consistent with *T. gondii* seroprevalence reported in similar studies worldwide, where *T. gondii* seroprevalence varied widely across different geographical areas [38]. Logistic regression revealed geography as the principal risk factor, with Diqing exhibiting the highest odds of infection (27.4%; OR = 3.62, 95% CI: 2.41–5.44, *p* < 0.001), probably reflecting divergent climates, culinary practices, economic development, hygiene habits, and regional control policies. Humid climates with large diurnal temperature swings, semi-nomadic herding and raw-meat consumption create optimal conditions for oocyst survival and parasite transmission. Wenshan (17.3%), with warm temperatures and abundant wildlife, and the southern humid prefectures of Pu’er, Baoshan and Dehong (15–17%) also registered elevated prevalences. High rainfall (~20 °C mean annual temperature) facilitated prolonged oocyst sporulation, while traditional raw-meat dishes established a bidirectional “human–cat–livestock” transmission loop that accelerated parasite circulation.

This study has several limitations. First, detailed metadata, including management practices, cat exposure, age, sex, and production system were largely unavailable, and the cross-sectional design precludes causal inference. Second, the reliance on serology (MAT) without molecular confirmation reflects exposure rather than active infection. Third, poultry species were grouped together, which may mask species-specific differences. Fourth, the interpretations regarding ecological and climatic drivers remain exploratory, as they lack support from direct environmental measurements. In light of these limitations, future studies in Yunnan Province should integrate molecular methods, collect comprehensive risk-factor data, and adopt longitudinal designs to better elucidate the transmission dynamics of *T. gondii*.

In summary, our study represents the first large-scale comprehensive analyses of *T. gondii* seroprevalence in Yunnan’s livestock and poultry and associated risk factors, which may provide baseline information for the development of control measures against infection in domesticated animals in Yunnan Province. However, there were some limitations in our study. For example, some samples lacked detailed information such as age, gender, management mode, and presence of cats, thus detailed analyses of the effect of these factors on *T. gondii* seroprevalence could not be performed.

## 5. Conclusions

This study provides the first provincial serological insights into *T. gondii* seroprevalence in livestock and poultry in Yunnan Province, China. A total of 10,766 serum samples were collected and screened; the overall seroprevalence was 13.7% (1474/10,766; 95% CI: 13.16–14.44%), with regions, season, and host species being identified as key risk factors. These findings confirm that Yunnan remains a meso-endemic region for *T. gondii*. Our results provide critical baseline data for elucidating transmission dynamics of *T. gondii* and designing targeted control strategies against *T. gondii*. They also offer an evidence-based reference for livestock development, food-safety assessment, and the inter-sectoral One-Health approach involving public-health authorities, wildlife agencies, and the broader research community.

## Figures and Tables

**Table 1:** Analysis of *T. gondii* seroprevalence and risk factors in livestock and poultry across 16 prefectures/cities in Yunnan Province, China.

Factors	Category	Samples	No. Positive	Positive Rate (95% CI)	*p* Value	Odds Ratio (OR, 95% CI)
Classification	pigs	**2954**	**446**	**15.1 (13.81–16.39%)**	**<0.001**	**2.49 (2.12–2.93)**
	cattle	**1950**	**252**	**12.9 (11.43–14.41%)**	**<0.001**	**2.08 (1.73–2.50)**
	sheep and goats	**1961**	**516**	**26.3 (24.36–28.26%)**	**<0.001**	**5.00 (4.26–5.87)**
	poultry	3901	260	6.7 (5.88–7.45%)		Reference
Season	Spring	3141	327	10.4 (9.39–11.53%)		Reference
	Summer	**927**	**155**	**16.7 (14.46–19.26%)**	**<0.001**	**1.73 (1.41–2.13)**
	Autumn	3795	431	11.4 (10.39–12.41%)	0.209	1.10 (0.95–1.28)
	Winter	**2903**	**561**	**19.3 (17.93–20.80%)**	**<0.001**	**2.06 (1.78–2.39)**
Region	Xishuangbanna	728	73	10.0 (8.05–12.42%)	0.706	1.07 (0.75–1.52)
	Baoshan	**513**	**81**	**15.8 (12.89–19.20%)**	**<0.001**	**1.80 (1.27–2.54)**
	Chuxiong	910	112	12.3 (10.33–14.60%)	0.068	1.35 (0.98–1.86)
	Dali	930	106	11.4 (9.51–13.60%)	0.201	1.37 (1.01–1.87)
	Dehong	**421**	**64**	**15.2 (12.09–18.95%)**	**<0.01**	**1.72 (1.19–2.48)**
	Diqing	**515**	**141**	**27.4 (23.71–31.39%)**	**<0.001**	**3.62 (2.63–4.97)**
	Honghe	710	67	9.4 (7.50–11.81%)		Reference
	Kunming	**701**	**115**	**16.4 (13.85–19.33%)**	**<0.001**	**1.90 (1.38–2.61)**
	Lijiang	433	45	10.4 (7.86–13.62%)	0.598	1.14 (0.78–1.68)
	Lincang	387	42	10.9 (8.13–14.35%)	0.454	1.17 (0.78–1.76)
	Nujiang	**693**	**96**	**13.9 (11.48–16.62%)**	**0.01**	**1.54 (1.11–2.15)**
	Puer	**813**	**128**	**15.7 (13.40–18.41%)**	**<0.001**	**1.79 (1.31–2.46)**
	Qujing	**635**	**89**	**14.0 (11.53–16.93%)**	**<0.01**	**1.56 (1.12–2.19)**
	Wenshan	**603**	**104**	**17.3 (14.44–20.47%)**	**<0.001**	**2.00 (1.44–2.78)**
	Yuxi	637	77	12.1 (9.78–14.85%)	0.117	1.28 (0.91–1.81)
	Zhaotong	1137	134	11.8 (10.04–13.79%)	0.116	1.32 (0.98–1.80)
Total		10,766	1474	13.7 (13.05–14.35%)		

Note: Seasons are defined as spring (March–May), summer (June–September), autumn (October–November), and winter (December–February). Bold values indicate statistical significance at the *p* < 0.05 level.

**Table 2:** Analyses of risk factors for *T. gondii* infection in different species in Yunnan Province, China.

Factors	Category	Samples	No. Positive	Positive Rate (95% CI)	*p* Value	Odds Ratio (95% CI)
**Swine**						
Overall		2954	446	15.1 (13.85–16.43)		
Gender	female	246	35	14.2 (10.41–19.14)		Reference
	male	321	58	18.1 (14.25–22.65)	0.222	1.33 (0.84–2.10)
	unrecorded	2377	353	14.9 (13.48–16.34)	0.793	1.05 (0.72–1.53)
Age	≥12 months	247	47	19.0 (14.62–24.38)	0.101	1.45 (0.93–2.26)
	<12 months	330	46	13.9 (10.62–18.09)		Reference
	unrecorded	2377	353	14.9 (13.48–16.34)	0.662	1.08 (0.77–1.50)
Season	Spring	845	110	13.0 (10.92–15.46)	0.140	1.24 (0.95–1.61)
	Summer	**192**	**55**	**28.7 (22.72–35.41)**	**<0.01**	**3.32 (2.32–4.74)**
	Autumn	1361	147	10.8 (9.26–12.56)		Reference
	Winter	**556**	**134**	**24.1 (20.73–27.83)**	**<0.01**	**2.62 (2.02–3.40)**
**Cattle**						
Overall		1950	252	12.9 (11.51–14.49)		
Gender	female	164	23	14.0 (9.53–20.17)	0.242	1.43 (0.79–2.58)
	male	263	27	10.3 (7.15–14.52)		Reference
	unrecorded	1513	202	13.4 (11.73–15.16)	0.170	1.35 (0.88–2.06)
Age	≥12 months	362	38	10.5 (7.74–14.08)		Reference
	<12 months	75	12	16.0 (9.40–25.92)	0.176	1.62 (0.80–3.28)
	unrecorded	1513	202	13.4 (11.73–15.16)	0.145	1.31 (0.91–1.90)
Season	Spring	**490**	**71**	**14.5 (11.65–17.88)**	**<0.01**	**1.64 (1.14–2.34)**
	Summer	216	26	12.0 (8.35–17.05)	0.257	1.32 (0.82–2.14)
	Autumn	**551**	**90**	**16.3 (13.48–19.65)**	**<0.01**	**1.89 (1.34–2.65)**
	Winter	693	65	9.4 (7.43–11.78)		Reference
**Sheep and goats**						
Overall		1961	516	26.3 (24.41–28.31)		
Gender	female	223	45	20.2 (15.44–25.93)		Reference
	male	339	81	23.9 (19.66–28.71)	0.302	1.24 (0.82–1.87)
	unrecorded	**1399**	**390**	**27.9 (25.59–30.28)**	**<0.05**	**1.53 (1.08–2.16)**
Age	≥12 months	443	102	23.0 (19.35–27.17)	0.507	1.18 (0.72–1.95)
	<12 months	119	24	20.2 (13.94–28.26)		Reference
	unrecorded	1399	390	27.9 (25.59–30.28)	0.072	1.53 (0.96–2.43)
Season	Spring	315	59	18.7 (14.81–23.41)		Reference
	Summer	**147**	**54**	**36.7 (29.37–44.77)**	**<0.01**	**2.52 (1.63–3.91)**
	Autumn	685	146	21.3 (18.41–24.54)	0.347	1.18 (0.84–1.65)
	Winter	**814**	**257**	**31.6 (28.47–34.85)**	**<0.01**	**2.00 (1.46–2.76)**
**Poultry**						
Overall		3901	260	6.7 (5.92–7.49)		
	**female**	**2657**	**193**	**7.26 (6.34–8.31)**	**<0.01**	**1.50 (1.10–2.05)**
	male	1089	54	5.0 (3.82–6.41)		Reference
	<90 d	77	8	10.4 (5.36–19.18)		2.11 (0.94–4.69)
	90–180 d	728	38	5.2 (3.83–7.08)		Reference
	>180 d	3096	214	6.91 (6.07–7.86)	0.099	1.35 (0.95–1.92)
Feeding model	Free-range	1287	56	4.4 (3.37–5.61)		Reference
	**Intensive**	**2614**	**204**	**7.8 (6.84–8.90)**	**<0.01**	**1.86 (1.37–2.52)**
Season	**Spring**	**1491**	**87**	**5.8 (4.75–7.14)**	**<0.05**	**1.49 (1.04–2.13)**
	Summer	372	20	5.4 (3.51–8.16)	0.259	1.36 (0.80–2.33)
	Autumn	1198	48	4.0 (3.04–5.27)		Reference
	**Winter**	**840**	**105**	**12.5 (10.43–14.91)**	**<0.01**	**3.42 (2.40–4.88)**

Bold values indicate statistical significance at the *p* < 0.05 level.

## Data Availability

The data presented in this study are included in the article. Further inquiries can be directed to the corresponding author.

## Supplementary Materials

The following supporting information can be downloaded at: https://www.mdpi.com/article/10.3390/vetsci13060517/s1, Table S1: Sample collection information by site and species; Table S2: Regional risk factors associated with *T. gondii* seroprevalence in livestock and poultry in Yunnan Province, China.
