# Supplementary material for: Seroprevalence of Toxoplasma gondii in Livestock and Poultry in Yunnan Province, China: A Cross-Sectional Study
Source: Vet Sci. 2026 May 27;13(6):517. doi: 10.3390/vetsci13060517 (PMC13307748; doi:10.3390/vetsci13060517)
Supplement: Supplementary file 1 [file vetsci-13-00517-s001.zip › vetsci-4332019-supplementary.pdf]

Supplementary Material

Supplementary table S1: Sample collection information by site and species

Sample sites	pigs	cattle	sheep and goats	poultry	Total
Xishuangbanna	168	179	93	288	728
Baoshan	131	90	112	180	513
Chuxiong	208	83	131	488	910
Dali	264	294	161	211	930
Dehong	143	123	45	110	421
Diqing	233	110	62	110	515
Honghe	237	42	76	355	710
Kunming	142	265	186	108	701
Lijiang	163	68	92	110	433
Lincang	139	72	66	110	387
Nujiang	324	99	160	110	693
Puer	131	154	160	368	813
Qujing	141	62	237	195	635
Wenshan	177	144	87	195	603
Yuxi	171	85	178	203	637
Zhaotong	182	80	115	760	1137
Total	2954	179	93	288	10766

Supplementary table S2: Regional risk factors associated with *T. gondii* seroprevalence in livestock and poultry in Yunnan Province, China.

Factors	Category	Samples	No. Positive	Positive Rate (95% CI)	*p* Value	Odds Ratio (95% CI)
Swine						
Overall		2954	446	15.1（13.85-16.43）		
Region	Xishuang banna	**168**	**17**	**10.1（6.41-15.61）**	**<0.01**	**1.37（0.64-2.91）**
	Baoshan	**131**	**17**	**13.0（8.26-19.80）**	**<0.05**	**1.81（0.85-3.88）**
	Chuxiong	208	29	13.9（9.89-19.31）	0.69	1.97（0.99-3.92）
	Dali	264	34	12.9（9.36-17.46）	0.16	1.80（0.92-3.51）
	Dehong	**143**	**26**	**18.2（12.72-25.31）**	**<0.01**	**2.70（1.33-5.48）**
	Diqing	**233**	**77**	**33.1（27.33-39.32）**	**<0.01**	**6.00（3.20-11.24）**
	Honghe	237	28	11.8（8.30-16.55）	0.166	1.63（0.82-3.25）
	Kunming	**142**	**25**	**17.6（12.22-24.70）**	**<0.01**	**2.60（1.28-5.29）**
	Lijiang	163	15	9.2（5.66-14.63）	0.598	1.23（0.57-2.68）
	Lincang	**139**	**22**	**15.8（10.69-22.80）**	**<0.05**	**2.29（1.11-4.72）**
	Nujiang	324	41	12.7（9.47-16.72）	0.090	1.76（0.92-3.39）
	Puer	**131**	**21**	**16.0（10.73-23.27）**	**<0.05**	**2.32（1.12-4.83）**
	Qujing	141	19	13.5（8.80-20.09）	0.093	1.89（0.90-3.98）
	Wenshan	**177**	**29**	**16.4（11.66-22.54）**	**<0.05**	**2.38（1.19-4.76）**
	Yuxi	171	13	7.6（4.50-12.57）		Reference
	Zhaotong	**182**	**33**	**18.1（13.21-24.37）**	**<0.01**	**2.69（1.36-5.31）**
Cattle						
Overall		1950	252	12.9（11.51-14.49）		
Region	Xishuang banna	179	15	8.4（5.14-13.36）	0.189	2.74（0.61-12.36）
	Baoshan	90	7	7.8（3.82-15.19）	0.257	2.53（0.51-12.61）
	Chuxiong	83	10	12.1（6.68-20.78）	0.075	4.11（0.87-19.48）
	Dali	**294**	**45**	**15.3（11.64-19.87）**	**<0.05**	**5.42（1.28-22.98）**
	Dehong	**123**	**17**	**13.8（8.81-21.02）**	**<0.05**	**4.81（1.08-21.54）**
	Diqing	**110**	**22**	**20.0（13.60-28.43）**	**<0.01**	**7.50（1.70-33.09）**
	Honghe	42	3	7.1（2.46-19.01）	0.372	2.31（0.37-14.44）
	Kunming	**265**	**49**	**18.5（14.28-23.60）**	**<0.05**	**6.81（1.61-28.80）**
	Lijiang	68	8	11.8（6.08-21.54）	0.088	4.00（0.82-19.62）
	Lincang	72	4	5.6（2.18-13.43）	0.521	1.77（0.31-9.98）
	Nujiang	99	7	7.1（3.47-13.88）	0.313	2.28（0.46-11.36）
	Puer	**154**	**24**	**15.6（10.70-22.14）**	**<0.01**	**5.54（1.27-24.20）**
	Qujing	62	2	3.2（0.89-11.02）		Reference
	Wenshan	144	17	11.8（7.50-18.09）	0.069	4.02（0.90-17.94）
	Yuxi	85	10	11.8（6.52-20.32）	0.081	4.00（0.84-18.95）
	Zhaotong	**80**	**12**	**15.0（8.79-24.41）**	**<0.01**	**5.29（1.14-24.61）**

Supplementary table S2: continued

Factors	Category	Samples	No. Positive	Positive Rate (95% CI)	*p* Value	Odds Ratio (95% CI)
Sheep and goats						
Overall		1961	516	26.3（24.41-28.31）		
Region	Xishuang banna	**93**	**31**	**33.3（24.58-43.41）**	**<0.01**	**4.53（2.31-8.22）**
	Baoshan	**112**	**42**	**37.5（29.09-46.74）**	**<0.01**	**5.44（2.86-10.34）**
	Chuxiong	131	22	16.8（11.36-24.12）	0.086	1.83（0.92-3.65）
	Dali	161	16	9.9（6.21-15.53）		Reference
	Dehong	**45**	**16**	**35.6（23.22-50.16）**	**<0.01**	**5.00（2.25-11.12）**
	Diqing	**62**	**27**	**43.6（31.94-55.91）**	**<0.01**	**6.99（3.40-14.37）**
	Honghe	**76**	**21**	**27.6（18.84-38.58）**	**<0.01**	**3.46（1.68-7.11）**
	Kunming	186	29	15.6（11.08-21.49）	0.121	1.67（0.87-3.21）
	Lijiang	92	14	15.2（9.29-23.94）	0.215	1.63（0.75-3.51）
	Lincang	66	10	15.2（8.44-25.69）	0.266	1.62（0.69-3.78）
	Nujiang	**160**	**37**	**23.1（17.27-30.24）**	**<0.01**	**2.73（1.45-5.14）**
	Puer	**160**	**48**	**30.0（23.44-37.50）**	**<0.01**	**3.88（2.10-7.20）**
	Qujing	**237**	**54**	**22.78（17.90-28.53）**	**<0.01**	**2.67（1.47-4.87）**
	Wenshan	**87**	**45**	**51.72（41.37-61.93）**	**<0.01**	**9.71（4.99-18.90）**
	Yuxi	**178**	**43**	**24.16（18.46-30.95）**	**<0.01**	**2.89（1.55-5.37）**
	Zhaotong	**115**	**61**	**53.04（43.97-61.92）**	**<0.01**	**10.24（5.44-19.28）**
Poultry						
Overall		3901	260	6.7（5.92-7.49）		
Region	Xishuang banna	288	10	3.5（1.90-6.27）		Reference
	Baoshan	**180**	**15**	**8.3（5.11-13.29）**	**<0.05**	**2.53（1.11-5.76）**
	Chuxiong	**488**	**51**	**10.5（8.04-13.48）**	**<0.01**	**3.24（1.62-6.50）**
	Dali	211	11	5.2（2.94-9.09）	0.342	1.53（0.64-3.67）
	Dehong	110	5	4.6（1.96-10.20）	0.616	1.32（0.44-3.96）
	Diqing	**110**	**15**	**13.6（8.44-21.29）**	**<0.01**	**4.39（1.91-10.10）**
	Honghe	355	15	4.2（2.58-6.85）	0.624	1.23（0.54-2.77）
	Kunming	**108**	**12**	**11.1（6.47-18.42）**	**<0.01**	**3.48（1.46-8.30）**
	Lijiang	110	8	7.3（3.73-13.70）	0.110	2.18（0.84-5.68）
	Lincang	110	6	5.5（2.52-11.39）	0.372	1.60（0.57-4.52）
	Nujiang	**110**	**11**	**10.0（5.68-17.02）**	**<0.01**	**3.09（1.27-7.50）**
	Puer	**368**	**35**	**9.5（6.92-12.94）**	**<0.01**	**2.92（1.42-6.01）**
	Qujing	195	14	7.2（4.32-11.69）	0.072	2.15（0.94-4.95）
	Wenshan	195	13	6.7（3.94-11.07）	0.112	1.99（0.85-4.62）
	Yuxi	203	11	5.4（3.05-9.44）	0.298	1.59（0.66-3.82）
	Zhaotong	760	28	3.7（2.56-5.27）	0.870	1.06（0.51-2.22）
